# Small Molecule Myeloperoxidase (MPO) Inhibition Prevents Delayed Cerebral Injury (DCI) After Subarachnoid Hemorrhage (SAH) in a Murine Model

**DOI:** 10.1007/s12028-024-02169-x

**Published:** 2024-12-10

**Authors:** Safiye Limon, Aminata P. Coulibaly, Jose Javier Provencio

**Affiliations:** 1 Department of Neurology, University of Virginia, Charlottesville, VA 22908 USA; 2 Department of Neuroscience, West Virginia University, Morgantown, WV 26506 USA

**Keywords:** Subarachnoid hemorrhage, Delayed cerebral injury, Inflammation, Neutrophils, Myeloperoxidase, Memory

## Abstract

**Background:**

Delayed cerebral injury (DCI) after aneurysmal subarachnoid hemorrhage (SAH) is a preventable injury that would improve patient outcomes if an effective treatment can be developed. The most common long-term disability in patients with SAH is cognitive dysfunction. Contrary to the common theory that damage from DCI originates solely from ischemia caused by cerebral vasospasm, inflammation has been shown to be an important independent mediator of DCI.

**Methods:**

Neutrophil infiltration of the meninges is a critical step in developing late spatial memory deficits in a murine model of SAH and may serve as a surrogate marker for disease progression. Importantly, myeloperoxidase (*MPO*) null mice do not develop meningeal neutrophilia and are protected from spatial memory deficits.

**Results:**

In this study, wildtype mice administered a single dose of the MPO inhibitor (MPOi) AZD5904 at peak neutrophil entry day have a higher percentage of neutrophils that remain in the meningeal blood vessel 6 days after the hemorrhage suggesting neutrophil extravasation into the meninges is inhibited (79 ± 20 vs. 28 ± 24, *p* < 0.01). Interestingly, the intraperitoneal route of administration has a larger effect than the intrathecal route suggesting that MPO inhibition is best administered systemically not in the central nervous system. Second, mice administered AZD5904 intraperitoneal for 4 consecutive days starting 2 days after the hemorrhage do not develop delayed spatial memory dysfunction (two-way analysis of variance, *p* > 0.001 *F* [2, 22] = 10.11).

**Conclusions:**

Systemic MPOi prevents neutrophil entry into the meninges and prevents spatial memory dysfunction. MPOi is a promising strategy for translation to patients with aneurysmal SAH.

**Supplementary Information:**

The online version contains supplementary material available at 10.1007/s12028-024-02169-x.

## Introduction

Delayed cerebral injury (DCI) affects up to 50% of patients with aneurysmal subarachnoid hemorrhage (SAH) and occurs between 4 and 12 days after the aneurysm rupture [1, 2]. DCI is associated with decreased level of consciousness, arterial vasculopathy (called cerebral vasospasm), and, occasionally, motor deficits. Patients who experience DCI experience impaired long-term memory function, fatigue, and decreased return to work [3–5]. Importantly, only a small proportion of patients who do not return to work have physical disability from stroke [3]. Therapies to prevent cerebral vasospasm have not improved outcome in patients with DCI (nimodipine, a Ca-channel blocker, improves outcome but does not impact vascular spasm) [6–8]. For this reason, our laboratory denotes DCI as “delayed cerebral injury” instead of “delayed cerebral ischemia.” The delay between the aneurysm rupture and onset of symptoms suggests that there is a window for treatment that may impact DCI outcome.

Contrary to the common theory that damage from DCI originates solely from ischemia caused by cerebral vasospasm, inflammation has been shown to be an important independent mediator of DCI [9–12]. Neutrophil activation in the meninges is a critical step to developing late spatial memory deficits in murine models of SAH [13–15]. Importantly, myeloperoxidase (*MPO*) null mice (*MPO* knockout [KO]) do not develop meningeal neutrophilia and are protected from spatial memory deficits suggesting that MPO inhibition may be a target for the prevention of DCI [16].

The MPO inhibitors (MPOi) are immunomodulators of inflammatory pathways that have neutrophils as a prominent component of the disease or injury. They have been tested in heart failure and atherosclerosis, but have also been studied in several neurological diseases [17, 18]. In this study, we investigate whether AZD5904, a potent MPOi, administered after the onset of SAH is a plausible agent for translation to human studies in patients with SAH.

## Methods

### Rationale

In this limited set of experiments, we tested the selective MPOi AZD5904 (MedChemExpress, Monmouth Junction, NJ) to determine its suitability for translation to human studies. Neutrophils play a critical role in the development of DCI, (Other leukocytes are less associated with the development of DCI) and in previous studies, selective neutrophil depletion after the hemorrhage appears to be sufficient to ameliorate DCI [14, 16, 19]. In addition, meningeal neutrophil infiltration is correlated with DCI in mice, and prevention of neutrophil entry is associated with amelioration of DCI memory deficits [16]. We therefore focused our efforts on neutrophils and inhibition of the neutrophil enzyme MPO.

**Fig. 1:**
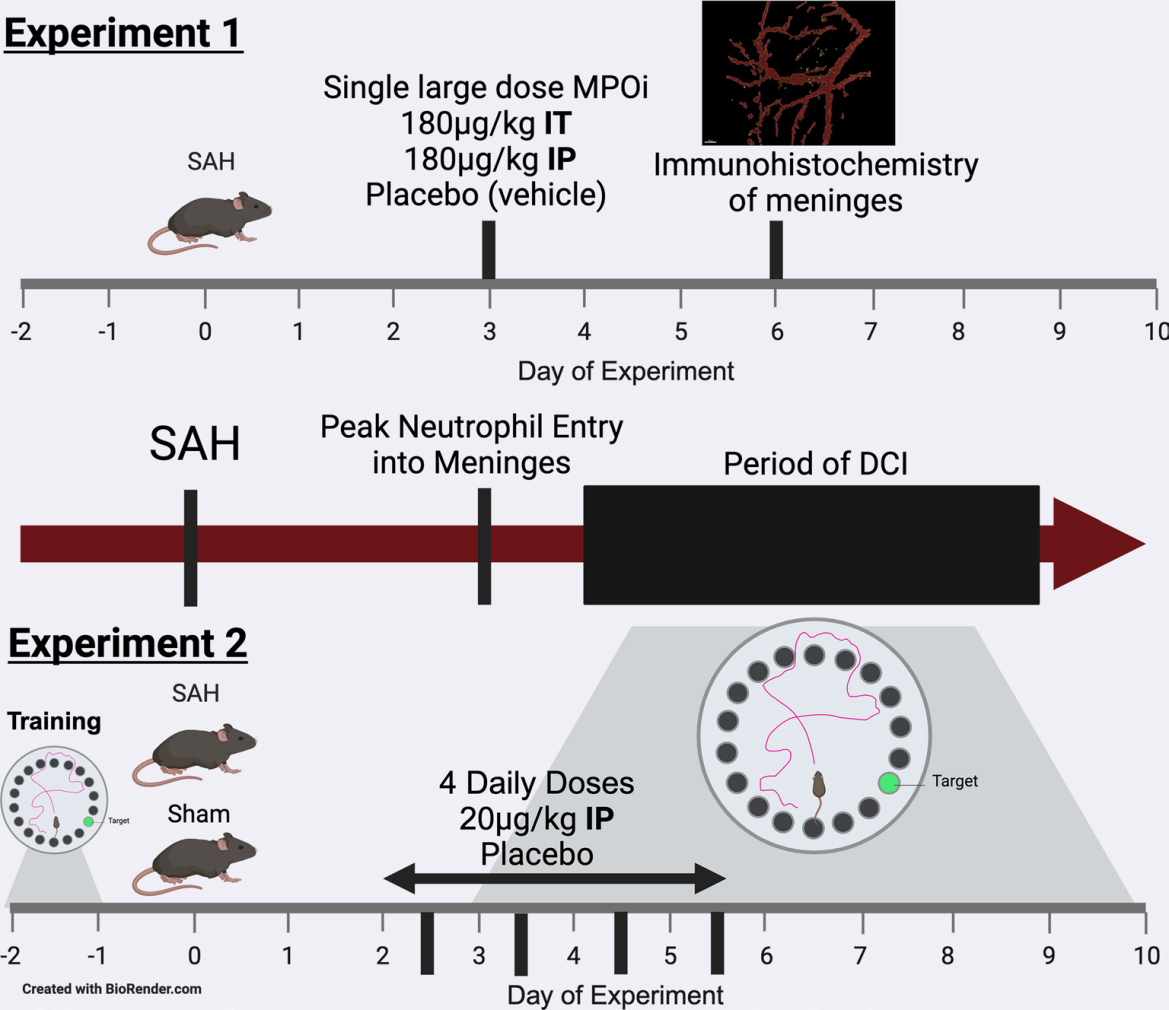
Experimental outline for the studies. The studies were devised to exploit the natural history of SAH where the hemorrhage is followed by a quiet period and the development to late complications that are driven by neutrophil derived MPO (middle timeline). The first set of experiments (Top figure) were designed to determine whether a large dose of the MPOi AZD5904 administered on the peak day of neutrophil extravasation into the meninges, inhibits neutrophil movement. The second set of experiments (Bottom figure) were devised to determine whether MPOi administered daily after the SAH reverses the spatial memory deficit associated with DCI. DCI, delayed cerebral injury, IP, intraperitoneal, IT, intrathecal, MPOi, myeloperoxidase inhibitor, SAH, subarachnoid hemorrhage

In the first experiment, we used the largest reported dose of AZD5904 (180 μg/kg) on the peak day of extravasation to determine whether blocking MPO impacts meningeal infiltration [16, 20]. In the second set of experiments, we used repeated dosing of AZD5904 at a lower dose (20 μg/kg) over 4 days (cumulative dose 80 μg/kg) based on mouse studies of MPO inhibition in other disease models to test if a therapy based on MPOi is a rational target for human testing [21]. We began administration after the hemorrhage (to make it more clinically relevant) and before peak meningeal infiltration (day 3: to increase the impact of the therapy and to mimic a human administration regimen). We chose the Barnes maze test of spatial memory because it has been validated in this model [14, 16]. We did not do a battery of behavioral tests because in previous studies, testing was congruent with the Barnes maze test [14]. The experimental strategy is outlined in Fig. 1.

### Mice and Surgical Treatments

All experiments were approved by the Animal Care and Use Committee at the University of Virginia. C57BL/6 J mice weighing 28–32 g and 10–12 weeks of age received analgesia before and after the procedure with bupivacaine (0.2 mg/kg) and buprenorphine (0.2 mg/kg), respectively. This model of SAH has been previously described [22]. In the prone position under anesthesia, using a surgical microscope, the posterior cervical muscles were dissected to reveal the atlanto-occipital membrane. A 30-gauge needle entered the membrane and transected a subarachnoid vein causing a hemorrhage. After closure with methacrylate (Vetbond; 3 M, St. Paul, MN), mice were recovered and returned to their cage. The sham procedure was identical except for the needle puncture and vein transection.

## Drug treatment

### Immunohistochemistry

Mice were randomly assigned to no treatment, a single dose of AZD5904 (180 μg/kg) through the intrathecal (IT) or intraperitoneal (IP) route 3 days after hemorrhage.

### Behavior

Mice were randomly assigned to receive either AZD5904 (20 µmol/kg) in dimethyl sulfoxide or phosphate buffered saline/dimethyl sulfoxide (placebo) IP. Mice began treatments 2 days after the hemorrhage/sham and continued daily for four doses. Daily doses were given 2 h after the completion of testing.

### Immunohistochemistry

Six days after SAH, the mice were euthanized, the skull cap was removed, and the meninges were dissected. The meninges were stained with monoclonal antibodies against CD31 (ab182981; Abcam, Cambridge, UK) and Ly6G-FITC (AB53453; Abcam, Cambridge, MA). Tiled images were obtained on the Olympus FV1200 confocal microscope with Fluoview software (Olympus, Tokyo, Japan). Each image was collected as a Z-stack. All images were converted to a maximum intensity image, by collapsing all stacks, reconstructed using “surface” and “spots” features using the Imaris software (Oxford Instruments, Abingdon, UK). Using the “distance from surface” feature of Imaris, the number of neutrophils observed at graded distances from the nearest blood vessel was obtained. Specifically, the distance from the center of the nearest blood vessel (the center point in line with the target neutrophil and side walls of the blood vessel measured as the (distal side wall position-proximal side wall position/2) to the neutrophil was measured for all neutrophils and plotted on a distance-incidence plot.

### Behavioral test

**Fig. 2:**
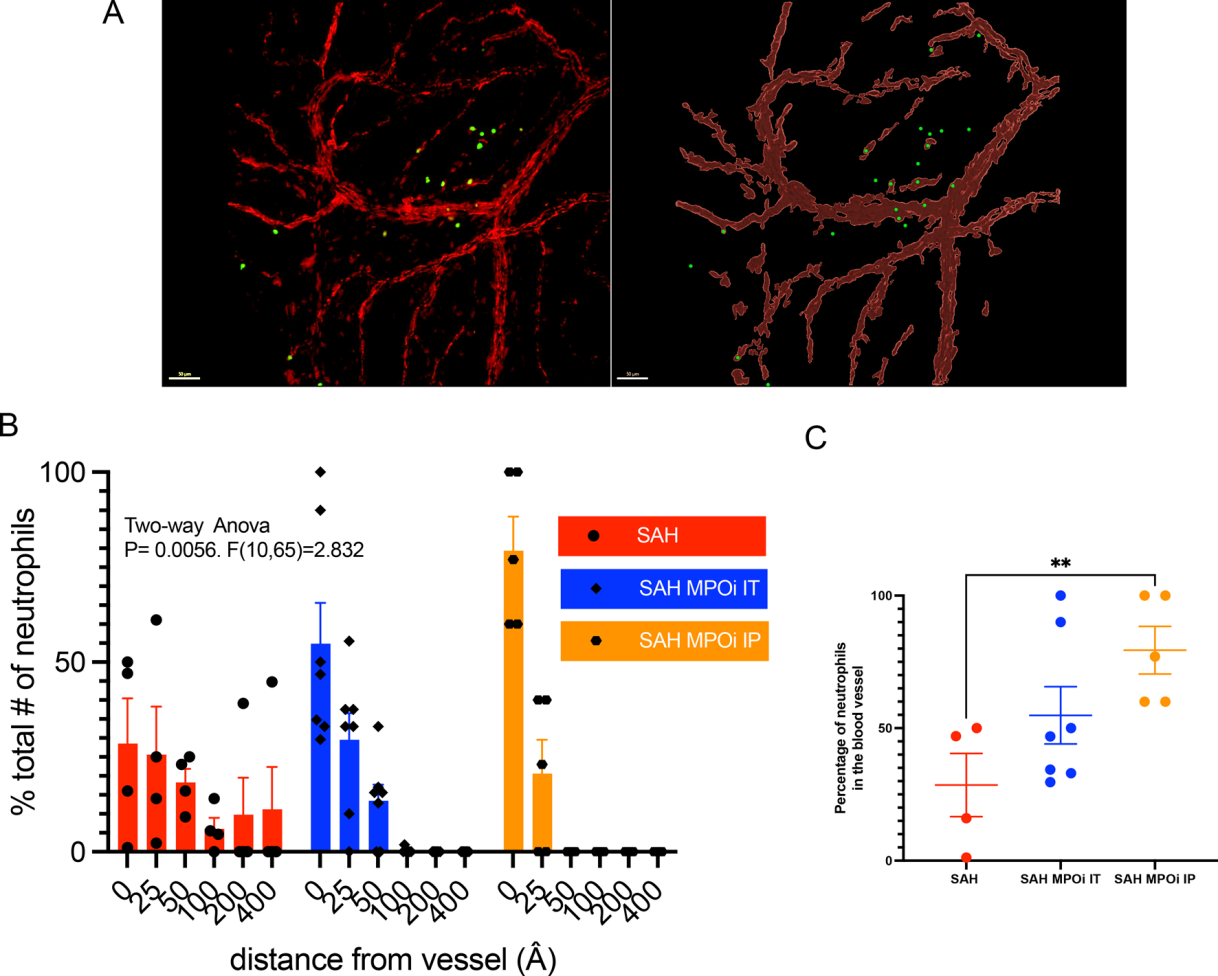
Neutrophils do not migrate out of blood vessels into the meninges after SAH with MPOi. **a** Quantitative analysis of neutrophils in the meninges comparing three groups: SAH with placebo, SAH with MPOi administered by IT injection, and SAH with MPOi administered through IP injection (example image and rendering). **b** Number of neutrophils by proximity to the nearest blood vessel measured in Å. There is a significant decrease in the distance neutrophils travel away from the blood vessel (0Å) in the IP MPOi compared with the placebo. **c,** The percentage of neutrophils found inside blood vessel (0 Å) across the same three categories: SAH, SAH MPOi IT, and SAH MPOi IP show a difference between SAH and SAH AZD5904 IP. Å, angstroms, IP, intraperitoneal, IT, intrathecal, MPOi, myeloperoxidase inhibitor, SAH, subarachnoid hemorrhage

The Barnes maze test is an elevated circular maze with 20 holes with a black escape box which did not change in location throughout the test. There are visual cues in the room to orient the test mice. The environment in the testing room included nondirect white light, high frequency (15,000 kHz) sound at 75 dB at a fixed source, and nondirect air movement. Starting each trial, a mouse is placed at the center of the maze. The trial was terminated at the point at which the mouse entered the box, stopped at the box for > 2 s, or did not find the box in 180 s. Mice were trained for 2 days before surgery to learn the placement of the escape box. Day 2 after surgery or sham, mice began daily testing until day 10 (Fig. 2a). Day 2 was considered a training day, and data collection started on day 3. Data were gathered using video tracking software (EthosVision; Noldus, Leesburg, VA). Parameters collected were latency to goal box, total distance traveled, heat maps of position, and mean velocity. Each animal underwent four trials each day. The mean performance on the trials each day was used for analysis.

### Statistics

Graphpad Prism 8.1 (Graphpad, La Jolla, CA) software was used to analyze all the data obtained. Scholl analysis was adaptive to evaluate the distance from the blood vessel to each of the neutrophils in the meninges [23]. The Mann–Whitney *U*-test and two-way analysis of variance (ANOVA) were used to determine significant differences between treatment groups (*p* < 0.05). No outliers were identified in the dataset by the robust regression and outlier removal method (ROUT).

For behavioral studies, our previous studies have shown that significant behavioral changes on the Barnes maze test by on the 10th day of the trial in mice after SAH compared with control are seen with six animals. The assessment was based on the following: Effect size 127 s, standard deviation 64.5 s, α 0.05, 0.90 with an expected sample size of 6. Therefore, a minimum of six animals was used for each experimental condition.

## Results

After SAH, peak neutrophil infiltration occurs 3 days after hemorrhage and is associated with memory changes [16]. *MPO* KO mice have fewer neutrophils in the parenchymal meninges (not in blood vessels) than wildtype mice and are protected from DCI, which is rescued by instillation of MPO. This first experiment tests if MPOi can prevent extravasation of neutrophils into the meningeal parenchyma similar to *MPO* KO mice and allows the testing of different routes of administration [16].

In this study, a single dose of AZD5904 administered at the peak day of extravasation (day 3) prevents neutrophils from exiting blood vessels to the parenchymal meninges. A representative image (Fig. 2a) shows the association with blood vessels and neutrophils in the source image (left) and the rendered image (right). The total number of neutrophils outside the blood vessel in the meninges (in the parenchyma) was lower in the MPOi IP group compared with the control group but did not meet the threshold for statistical significance (*p* = 0.055). Analysis of the distance of neutrophils to the nearest blood vessel shows that a larger proportion of neutrophils in the SAH group are farther from the blood vessel than in the MPOi IT and IP groups (two-way ANOVA, *p* < 0.05, *F* [10, 65] = 2.832) (Fig. 2b). The percentage of neutrophils that remain in the blood vessel (0 Å) and therefore do not extravasate into the parenchyma is significantly increased in MPOi IP compared with SAH alone (mean ± standard deviation 79 ± 20 vs. 28 ± 24, *p* < 0.05; Fig. 2c). Interestingly, the IP route worked best (the IT route was not significantly different than SAH), suggesting that inhibition of neutrophil extravasation in the blood is preferred. This supports the feasibility of administering AZD5904 in the blood of patients with SAH.

**Fig. 3:**
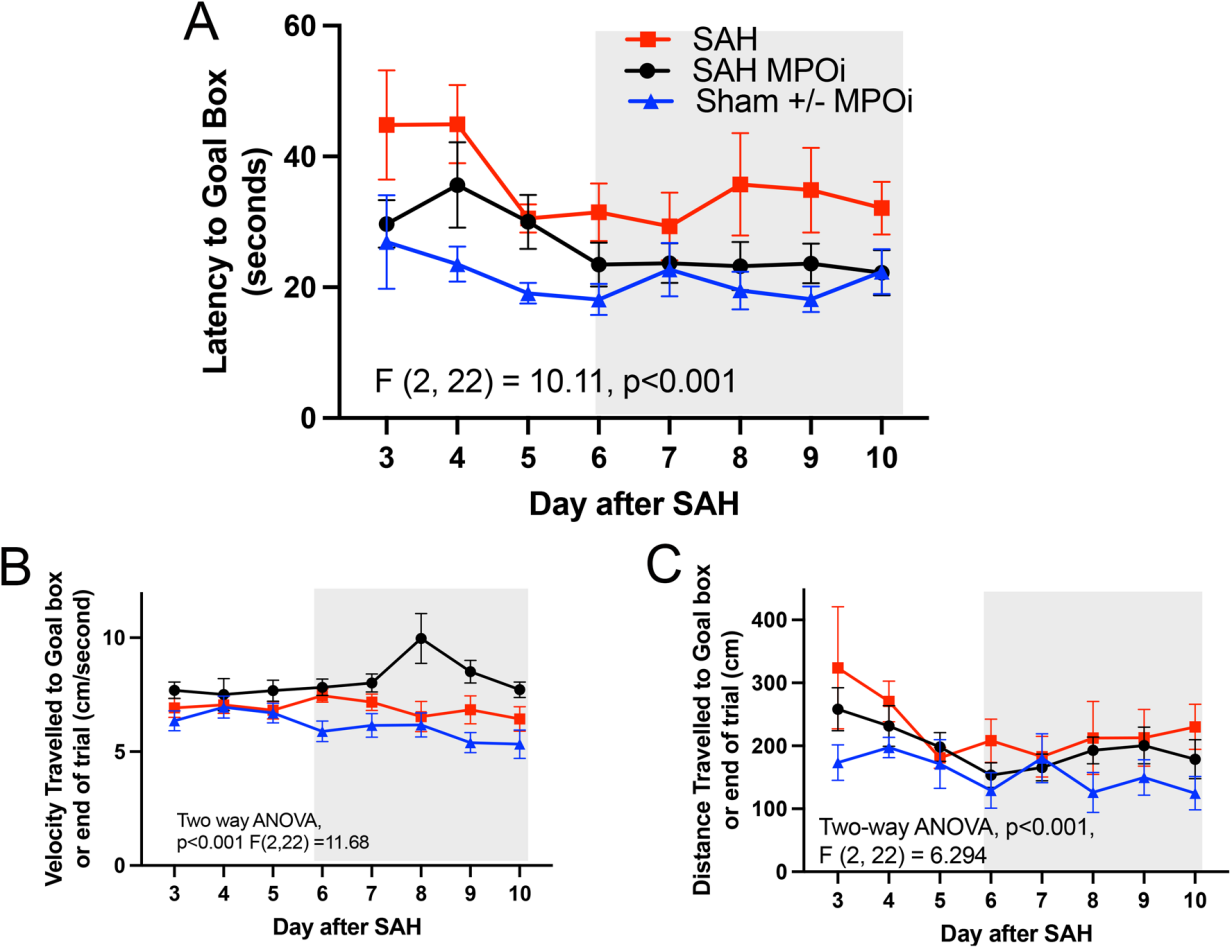
Barnes maze show improvement in latency to goal box in SAH after MPOi. After two days of training, mice received either SAH or sham surgery. Four doses of either MPOi or placebo were administered during days 2–5 while simultaneously undergoing the Barnes maze test. **a** Latency refers to the time to find the goal box. SAH placebo showed impaired spatial memory from day 6–10 (after drug course completed) compared with the sham placebo. SAH MPOi and sham placebo mice did not show impaired spatial memory. Importantly, MPOi rescued the impaired memory. **b** The mean velocity was significantly increased in MPOi treated SAH mice compared with the sham placebo and SAH. **c** There was no difference between mice in distance traveled to the goal box. ANOVA, analysis of variance, MPOi, myeloperoxidase inhibitor, SAH, subarachnoid hemorrhage

To test if MPO inhibition prevents the late long-term spatial memory impairment after SAH, we randomly assigned SAH and sham mice to receive MPOi IP or placebo (Fig. 3a). Four experimental conditions were tested: SAH, SAH MPOi IP (20 mM/kg AZD5904 given daily for 4 days, total dose 80 mM/Kg), sham, and sham MPOi IP. Sham and sham MPOi IP were similar (Supplemental Fig. 3b), so the sham and sham MPOi IP groups were combined (Fig. 3). Analysis of all four groups was conducted separately to ensure the results were consistent (Supplemental Fig. 1). Administration of MPOi in SAH animals significantly improved memory performance (subset two-way ANOVA, *p* > 0.001 *F* [2, 22]  = 10.11) (Fig. 2b). Performance between day 6 and 10 (after completion of the MPOi therapy), shows that treated SAH animals had performance similar to the sham mice (subset two-way ANOVA, *p* = 0.394 *F* [1, 11] = 0.7854) (Fig. 3b, gray box). The mean velocity traveled to find the goal box was significantly faster in the SAH MPOi group than control or SAH, with a peak at day 8 (two-way ANOVA, *p* < 0.001 *F* [2, 22] = 11.68) (Fig. 3c), and the distance traveled was significantly different between the three groups (two-way ANOVA, *p* < 0.001 *F *[2, 22] = 6.29), but there was no difference between SAH and SAH MPOi (Fig. 2d).

In a subset analysis of the Barnes maze test using sex as a variable, male and female mice with SAH without MPOi show a trend toward behaving differently (two-way ANOVA, *p* = 0.509, *F* [1, 3] = 9.986) (Supplemental Fig. 2a). The trend continued with MPOi but was not significantly different (Supplemental Fig. 2b). The behavior of SAH and sham mice without MPOi appear different between female and male mice with female differences more pronounced (two-way ANOVA, *p* < 0.01 F [1, 12] = 10.51) (Supplemental Fig. 2c and d). When comparing male and female mice with and without treatment, there was no difference, although the sample size was insufficient to conclude this definitively (Supplemental Fig. 2e and f).

## Discussion

The use of MPO as a target to treat DCI after SAH would herald a paradigm shift in therapy for patients with SAH. Previous treatment strategies focused on preventing vasospasm-associated with DCI in the hopes of preventing vasospasm-associated ischemia [6, 7]. More than physical disability, which would be expected in ischemic stroke, patients with DCI have cognitive deficits [3, 5, 24]. To improve patient outcomes, treatments must address cognitive deficits. In this study, we show that a small molecule MPOi, AZD5904, prevents the late development of memory deficits similar to the memory deficits that occur in patients with SAH and DCI. A strength of this work is that it characterizes the velocity and distance traveled by the mice in the spatial memory test as well as potential sex differences in the response to SAH. These may be important variables to consider for future mouse studies. Sex and correlates of the components functional memory may also be important variables in patients with SAH.

Inflammation as a cause of DCI after SAH has become a more popular theory in the last 20 years [10, 25, 26]. Unfortunately, the term “inflammation” is extremely broad and encompasses many pathways as different as early nonspecific innate immunity and adaptive immunity with long-term immunological memory. Understanding not just that inflammation is a causative agent, but also what specific pathways are involved, is critical. Here, we have focused on a subset of the innate immune system, the neutrophil response. Neutrophils, traditionally thought to be early, short-lived destructive cells that enter areas of infection and inflammation, have been found to do a multitude of other “jobs” in the right environment, ranging from phagocytosis to antigen presentation and cytokine release (reviewed in [27]). In SAH, neutrophils enter the central nervous system days after the initial hemorrhage and do not appear to act as destructive cells [15, 16].

Importantly, meningeal infiltration by neutrophils is a critical step in the development of DCI in this model and is decreased in *MPO* KO mice [16]. This allowed the use of meningeal inflammation as a surrogate to investigate the best route of administration of the drug. The single large dose of drug at the peak day of neutrophil extravasation was the most expedient way to address this. The decrease in percentage of neutrophils that leave the blood vessels and enter the meninges on day 3 after MPOi suggests that this agent functions in the same manner as the *MPO* KO mice experiments. Based on the finding that IP administration of AZD5904 works best to inhibit meningeal information, it appears that small molecule MPOi administered outside the blood–brain barrier is a viable target for clinical trials in patients with SAH.

All animal model experiments have limitations. This study was done in one center with one model; variables such as model effect and center effects could make this study less generalizable [28, 29]. Second, we used a single large dose of AZD5904 to investigate the optimal route of administration and smaller repeated doses in the behavioral test to recapitulate what is more likely to be used in a human trial. Although we found signals at all doses tested, there is still uncertainty about the optimal dose of the drug. Third, we have a limited understanding of how memory loss in mice recapitulate the patient experience in SAH. We chose to focus on a single cognitive test to more easily compare with previous studies in this model. A broader array of tests could have shown differences in more domains of memory and socialization. Because this study was focused on validating a small molecule inhibitor, we felt this was warranted. Cognitive studies of patients with SAH suggest that memory function is impaired, although true movement-modulated spatial memory (what is tested in Barnes maze) is not routinely tested in neuropsychological evaluations. Only multicenter animal trials using multiple models and human testing will answer these questions.

## Conclusions

Inhibition of myeloperoxidase through a small molecule inhibitor (AZD5904) improves spatial memory of mice experiencing experimental SAH. It appears that administration outside the blood–brain barrier is the optimal route. This compound holds promise as a target to treat or prevent DCI after aneurysmal SAH.

## Supplementary Information

Below is the link to the electronic supplementary material.

Supplementary file1 (PDF 724 KB)
