# Supplementary material for: Small Molecule Myeloperoxidase (MPO) Inhibition Prevents Delayed Cerebral Injury (DCI) After Subarachnoid Hemorrhage (SAH) in a Murine Model
Source: Neurocrit Care. 2024 Dec 10;42(3):945–52. doi: 10.1007/s12028-024-02169-x (PMC12137489; doi:10.1007/s12028-024-02169-x)
Supplement: Supplementary file 1 — Supplementary file1 (PDF 724 KB) [file 12028_2024_2169_MOESM1_ESM.pdf]

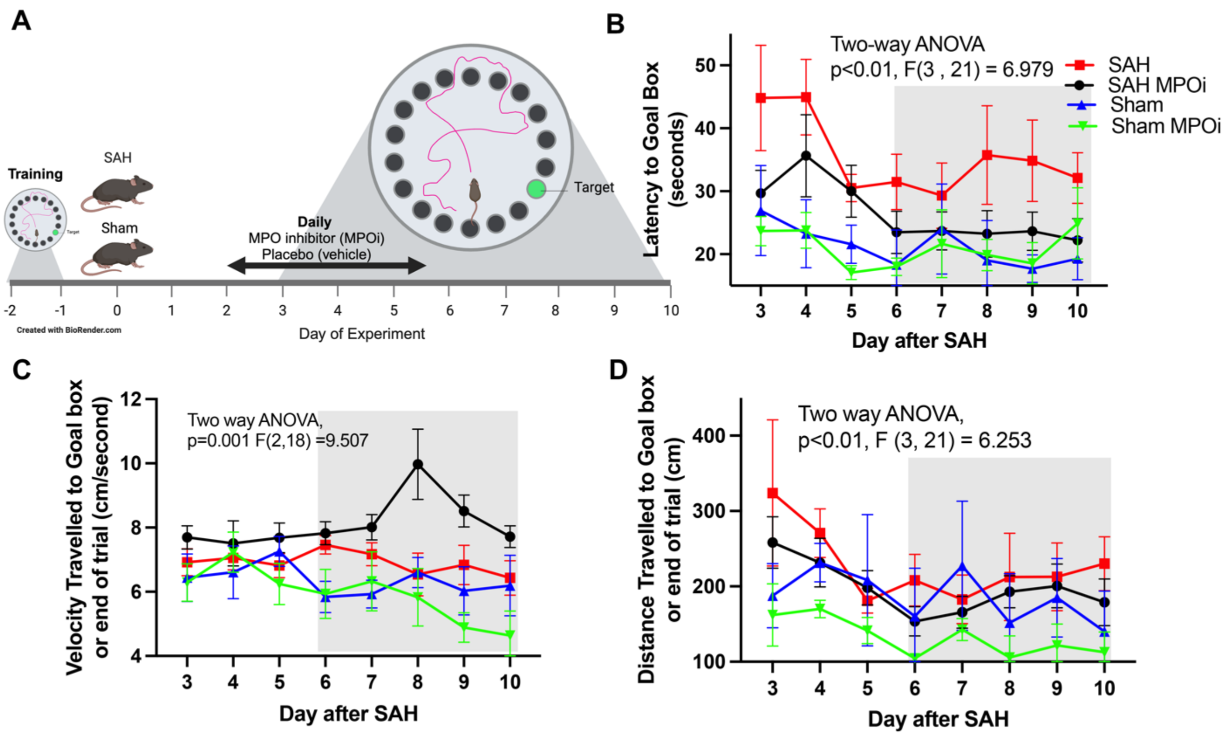

Supplemental Figure 1: Barnes maze analysis with Sham with MPOi group included. In Figure 2, the Sham MPOi group was removed from the graph to make the graph easier to visualize (although it was included in the analysis). This figure is the same information with the Sham MPOi group included.

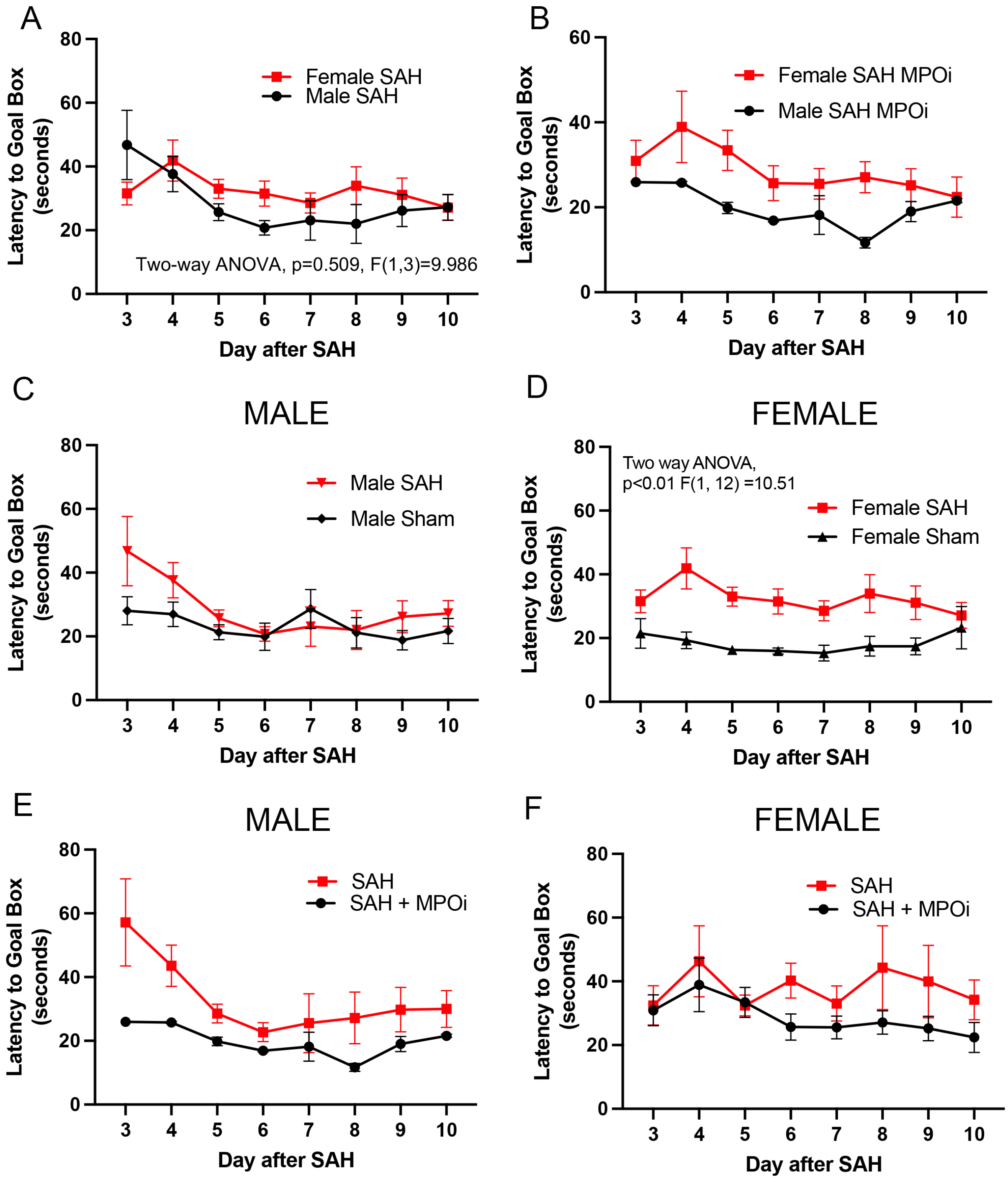

Supplemental Figure 2: Barnes maze latency to goal box differences between sexes.

Female vs. male latency to goal box regardless of treatment after subarachnoid hemorrhage (SAH) is not different **(A and B)**. When male and females without treatment

are investigated separately, there is a difference between SAH and sham groups in the responses of females but not males **(C and D)**. SAH animals with and without treatment did not have significant differences **(E and F)**. The difference in patterns suggests that the subset analysis may not have been powered to adequately see a difference.
